# Analysis of Anonymous Student Narratives About Experiences with Emergency Medicine Residency Programs

**DOI:** 10.5811/westjem.17973

**Published:** 2024-02-05

**Authors:** Molly Estes, Jacob Garcia, Ronnie Ren, Mark Olaf, Shannon Moffett, Michael Galuska, Xiao Chi Zhang

**Affiliations:** * Loma Linda University, Department of Emergency Medicine, Loma Linda, California; † Mount Sinai Morningside/West, Department of Emergency Medicine, New York, New York; ‡ University of Massachusetts, Department of Emergency Medicine, Boston, Massachusetts; § University of Florida Health Shands Hospital, Department of Emergency Medicine, Gainesville, Florida; ∥ Geisinger Commonwealth School of Medicine, Department of Emergency Medicine, Scranton, Pennsylvania; ¶ Rutgers New Jersey Medical School, Department of Emergency Medicine, Newark, New Jersey; # Conemaugh Memorial Medical Center, Department of Emergency Medicine, Johnstown, Pennsylvania; ** Thomas Jefferson University, Department of Emergency Medicine, Philadelphia, Pennsylvania

## Abstract

**Background:**

Academic emergency medicine (EM) communities have viewed anonymous online communities (AOC) such as Reddit or specialty-specific “applicant spreadsheets” as poor advising resources. Despite this, robust EM AOCs exist, with large user bases and heavy readership. Insights about applicants’ authentic experiences can be critical for applicants and program leadership decision-making. To date, there are no EM studies to qualitatively assess EM AOC narratives during the application cycle. Our goal was to perform a qualitative analysis of students’ EM program experiences through a publicly available AOC.

**Methods:**

This was a qualitative analysis of a publicly available, time-stamped, user-locked AOC dataset: “Official 2020–2021 Emergency Medicine Applicant Spreadsheet.” We extracted and then de-identified all data from selected sub-sheets entitled “Virtual Interview Impressions” and “Rotation Impressions.” Four investigators used constant comparative method to analyze the data inductively, and they subsequently met to generate common themes discussed by students. Preliminary thematic analysis was conducted on a random sample of 37/183 (20%) independent narratives to create the initial codebook. This was used and updated iteratively to analyze the entire narrative set consisting of 841 discrete statements. Finally, two unique codes were created to distinguish whether the identified sub-themes, or program attributes, were likely “modifiable” or “non-modifiable.”

**Results:**

We identified six major themes: living and working conditions; interpersonal relationships; learning experiences, postgraduate readiness, and online/virtual supplements. Common sub-themes included patient population (13%); resident personality (7%); program leadership personality (7%); relationship with faculty/leadership (6%); geography (4%); practice setting (4%); program reputation (4%), and postgraduate year-3 experiences (4%). Modifiable sub-themes outnumbered non-modifiable sub-themes, 60.7% to 39.3%.

**Conclusion:**

In this analysis of selected medical students’ narratives in an AOC, the majority of identified themes represented topics that may serve as external feedback for EM residency programs and their clerkships. Selective use of AOCs may set a precedent for future program assessments by applicants and inform program leadership of important programmatic elements in the eyes of applicants. It elucidates important themes in their interactions or learning experiences with programs and creates opportunities for learner-centric program improvement.

## INTRODUCTION

The academic community has traditionally viewed anonymous online communities (AOC) as poor resources for advising, recommending that students be wary of them or avoid them altogether.^1^ Common themes addressed against these forums include lack of commenter professionalism,^2^ ^–^ ^4^ information inaccuracy,^5^ and breach of ethics via malicious posting of falsified, incomplete, or privileged information.^2^ ^,^ ^3^ Prior studies have also shown that anonymous AOC commenters may not necessarily reflect the entire applicant population.^6^ Within emergency medicine (EM), students report information from AOCs such as Reddit and Student Doctor Net (SDN) as the “least trustworthy” compared to other advising resources.^7^

Despite this, most specialties, including EM, have robust AOCs for medical students, boasting large user bases and robust discussion threads with heavy readership.^8^ ^–^ ^11^ Within these anonymous forums, students discuss diverse topics about the application process, specialty-specific questions, and student experiences applying to, rotating at, or interviewing with specific programs.^12^ ^,^ ^13^ For example, studies identify “program-specific information” as a common theme in otolaryngology- and radiology-applicant AOCs; however, their findings were limited in characterizing specific topics discussed.^12^ ^,^ ^13^ There is also a consolidated, annually renewed, and user-generated Google Spreadsheet circulating within EM forums with a stated goal to “provide a central location for applicants to research different residency programs, view information about other applicants and where they are applying, and share information about away, interviews, and general advice.”^14^

For EM, the discussion of authentic, program-specific experiences, such as the student’s interview day experience and interaction with residents, have historically been ranked as the top two factors in impacting their rank order, making this information highly valuable to both applicants and program stakeholders.^15^ Our primary goal was to characterize what prospective EM applicants discuss in an AOC forum regarding their experiences with specific programs. Our secondary goal was to identify potentially useful information for program improvement and development.

## METHODS

This was a qualitative, retrospective review of a publicly available AOC for EM rotations in 2020–2021. It was submitted for institutional review board review through Thomas Jefferson University and determined to not meet the definition of human subjects research. We analyzed extracted data from an online, time-stamped, and user-locked Google Sheet entitled “Official 2020–2021 Emergency Medicine Applicant Spreadsheet,” whose link can be found within popular AOCs such as Reddit, SDN, and Discord.^14^ “The Spreadsheet” allows anonymous individuals to post requested information regarding specific EM programs. The spreadsheet contains multiple sub-pages, or “sheets,” to address different types of information an applicant might seek. This includes sheets listing program-specific facts such as “Program Benefits” and “Program Information”; sheets describing student experiences with a program like “Rotation Experience,” “Virtual IV (Interview) Impressions,” or “Name and Shame”; and sheets addressing miscellaneous application topics such as “Rejection/Wait List” or “Dropped Interviews” to help applicants coordinate logistics.

With permission from the page administrator, confirmed to be a current EM resident, we created a replica of the spreadsheet on September 12, 2021, for the purpose of this study. Upon review of all available sheets within the spreadsheet, the sub-pages entitled “Virtual IV” and “Rotation Impressions” were purposefully sampled via group consensus for analysis as they were felt to most likely include students’ direct impressions of programs. In contrast, we excluded sub-pages such as “Name and Shame” from qualitative analysis due to high likelihood of containing caustic and controversial narratives. As the purpose of this study was to investigate “what” is being said, not “who” is discussing them or to “whom” it is addressed, one investigator transferred all comments from the selected pages into a single dataset while removing potentially identifying user or program information.

We performed qualitative analysis primarily using the constant comparative method,^16^ where excerpts of raw data are organized into groups according to attributes and those groups are further structured to formulate a new theory. The selected sub-pages yielded 183 individual narratives discussing students’ impression of the de-identified programs. A random number generator was used to select 20% of individual narratives as a convenience sample for investigators to inductively create a working codebook, de novo. All duplicates were identified and removed, until the excerpts were all unique. The dataset was independently analyzed by the investigators [ME, JG, RR, XCZ] to identify thematic content within each narrative for inductive coding. Individually identified themes were compared among investigators to generate common themes. These themes were organized into major “themes” and “sub-themes” to create the initial codebook. This was used by a single investigator (JG) to code the entire dataset. Additional sub-themes identified during this process were updated into the codebook under existing major themes. Upon completion, all themes and their associated excerpts were reviewed by the remaining investigators to ensure coding consensus. The entire dataset was reviewed until no additional themes were identified (see Appendix 1).

Upon reviewing the final codebook, we created two additional thematic categories: 1) potentially “modifiable” program attributes and 2) less likely or “non-modifiable” program attributes. Drawing upon our collective experiences, we defined “modifiable” subthemes as attributes most likely under the direct control of the education leadership and “non-modifiable” subthemes as attributes that are either truly non-modifiable or would require significant input from outside stakeholders to change. This distinction was made with the understanding that different programs have different abilities to modify certain attributes.

## RESULTS

From 183 comments, 841 discrete statements were identified and coded. We identified six themes: working conditions; interpersonal relationships; learning experience; living conditions; postgraduate readiness; and online/virtual supplement, as shown in Figure and Table 1. The top two encoded themes—working conditions and interpersonal relationships—comprised 572 (68%) of the total coded statements (324 [38.5%] and 248 [29.5%], respectively).

**Table 1.** Identified themes with representative comments coded to each.

Theme	Representative comment
Working conditions	“…academic institution means a lot of consults sometimes, some 12-hour shifts (but mix of 8s and 12s), 50% of shifts as an intern are overnight…no debriefing process after codes/traumas…”
Interpersonal relationships	“Every program mentions family feel but having rotated here it was truly tight knit. Faculty and resident hang outs often including beach trips.”
Learning experience	“Most attendings tolerate students, and the rest are really proactive about teaching and getting the students involved…When it does settle a bit, residents are enthusiastic about your education for the most part. You’ll get to do almost any procedure you want because the residents have already done them a thousand times before.”
Living conditions	“Area right outside of [location] can be a pro or a con. Probably [would] have to deal with a lot of traffic and high cost of living.”
Postgraduate readiness	“Really old program, so alumni all over the country to help with job placement (last class 1/2 community, 1/4 fellowship, 1/4 academic). With 4 different hospitals, variety of training is very good and will be prepared for any type of job coming out.”
Online/virtual supplement	“Best ED tour, literally took a GoPro from the ED department to the actual ED so you could actually see the ED.”

**Figure.**
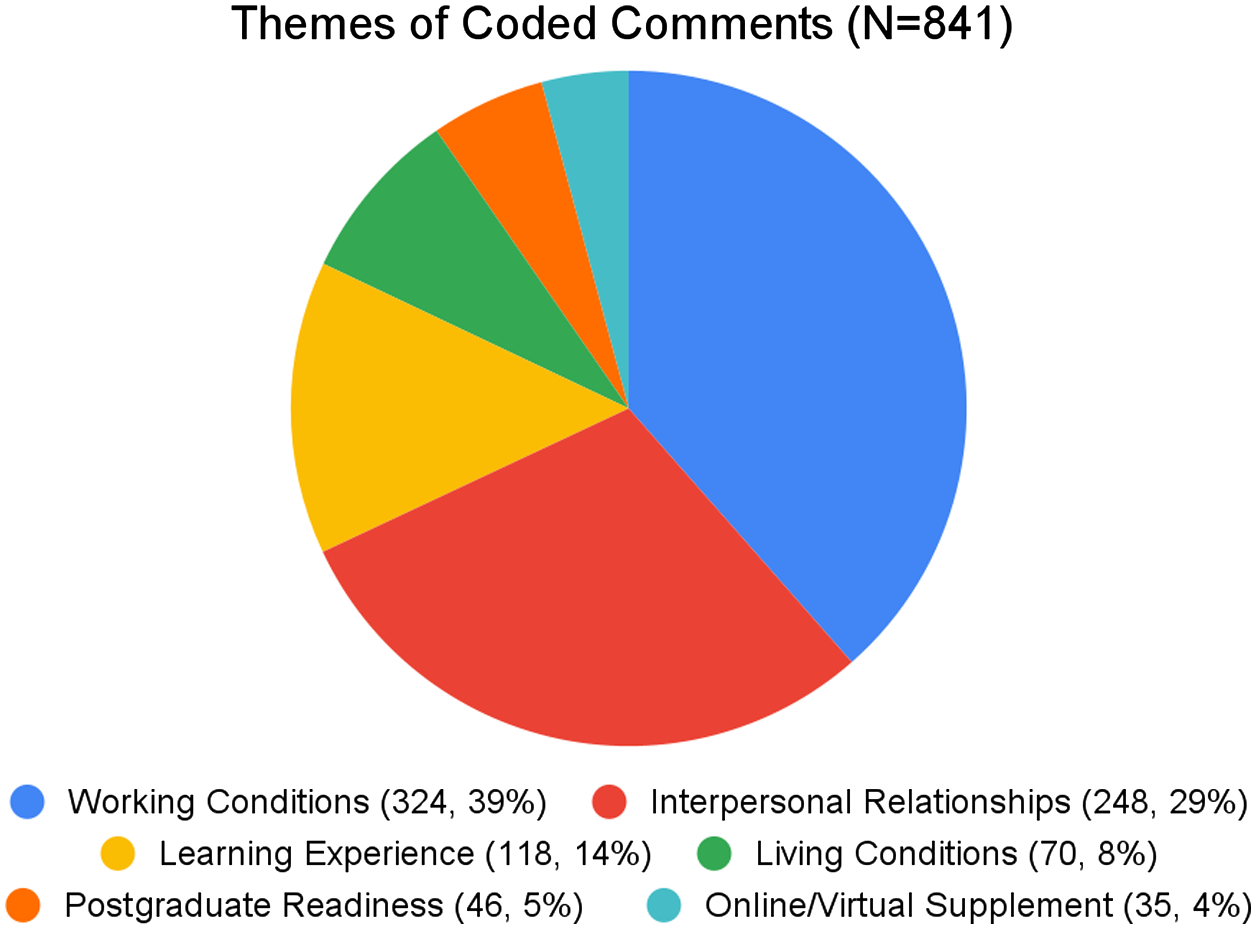
Thematic categories of coded statements, including the number of individual statements and percentage of total statements.

Sub-themes identified within each theme (see Appendix 1 for a full listing of sub-themes with their corresponding number of coded statements) were then subdivided to represent modifiable and non-modifiable clerkship/program aspects (Tables 2 and 3). Modifiable sub-themes outnumbered non-modifiable sub-themes (60.7% vs 39.3%). The sub-themes housed within the theme of interpersonal relationships represented the largest single category of modifiable attributes with 248 (29.5%) statements. Comments on working conditions and learning experience were the second and third largest categories, with 109 (13%) and 118 (14%) comments, respectively. The majority of non-modifiable sub-themes were found within the theme of working conditions with 215 (25.6%) individual comments, which represented 65% of all non-modifiable comments. The second largest non-modifiable sub-theme was within the theme of living conditions, including comments on the local geography, cost of living, or nearby amenities.

**Table 2.** Modifiable major themes and sub-themes determined by author consensus by a clerkship or residency program; percentages are of the total number of comments, N = 841.

Themes	Sub-themes	#	Total coded comments
Working conditions	Perks (funding for travel/activities, food, lounge, parking, etc)	37	109 (13.0%)
	DEI (includes LGBTQ+)	27	
	Relationship with other specialties	23	
	Wellness	20	
	Scutwork	2	
Interpersonal relationships	Residents	76	248 (29.5%)
	Other leadership/faculty personality	76	
	PD personality	56	
	Responsiveness to upward feedback	16	
	Opportunity for upward feedback	14	
	Generic	8	
	Objective experience	2	
Learning experience	Procedures	25	118 (14.0%)
	Didactics/conference	20	
	On-shift teaching	17	
	Autonomy	16	
	POCUS	12	
	Pediatric training	12	
	EMS/prehospital training	5	
	Scholarly tracks	5	
	Research	5	
	Personal patient load	1	
Online/virtual supplement	Virtual interview day	28	35 (4.2%)
	Virtual tour	4	
	Virtual rotation	2	
	Website	1	

*DEI*, diversity, equity, inclusion; *PD*, program director; *POCUS*, point-of-care ultrasound; *EMS*, emergency medical services.

**Table 3.** Non-modifiable major themes and sub-themes determined by author consensus by a clerkship or residency program; percentages are of the total number of comments, N = 841.

Themes	Sub-themes	#	Total coded comments
Working conditions	Patient population (underserved, volume, trauma, pathology etc)	66	215 (25.6%)
	Practice setting (community, academic, county, Lvl 1, HCA, etc)	66	
	Program reputation/prestige/age	21	
	Work hours	17	
	Ancillary healthcare staff	15	
	EHR	12	
	Salary	9	
	Metrics	6	
	Moonlighting	3	
Living conditions	Geography	53	70 (8.3%)
	Amenities	11	
	Cost of living	6	
Postgraduate readiness	Fellowships	17	46 (5.5%)
	Jobs	13	
	PGY4 experience (length of training)	12	
	PGY3 experience (length of training)	4	

*HCA*, Hospital Corporation of America; *EHR*, electronic health record; *PGY*, postgraduate year.

## DISCUSSION

Anonymous online communities have been historically viewed by clerkship and residency program leadership as unreliable forums for student discussion that foster confabulation of facts and operates on rumors and hearsay, a communication tool of the disgruntled, and not a resource to be taken seriously.^1^ ^–^ ^3^ ^,^ ^5^ ^–^ ^7^ This is the first study to describe, in detail, the narrative content of students discussing their program impressions on an AOC. Our findings suggest that many of the discussed items are common considerations for a student seeking to find the ideal next stage of training. Mentors in EM have historically encouraged prospective EM applicants to inquire about interpersonal interactions and resident working conditions within a specific program. Our analysis reveals that students are also seeking more information and commenting on many of the same factors we have been advising them to seek out.^17^

Moreover, analysis of the sub-themes reveals a unique trend toward potentially modifiable program attributes that, if addressed, could be mutually beneficial for programs and applicants. Topics such as perceived resident wellness, diversity, equity, and inclusion, opportunities for upward feedback, and effectiveness of on-shift teaching are all under the control of a program to potentially improve. Many of these topics are of rising importance to students.^18^ ^,^ ^19^ The availability of this information raises a very interesting question for programs and recruitment: if programs were aware of these discussed topics and the student comments relative to each topic, would a program be likely to change internal element(s) to make itself more appealing to students?^20^

In light of the recent National Resident Matching Program (NRMP) results from 2022 and the continued downtrend of applications in 2023,^21^ ^,^ ^22^ many EM programs must contend with a smaller applicant pool, which reduces the likelihood of filling programs, and overall program competitiveness for applicant recruitment. While we cannot predict future trends, our specialty has faced declining student applications for two years in a row with a rising number of residency programs and positions over the last several years. As traditional matching patterns begin to falter, residency leadership should consider addressing critical elements from AOCs, instead of ignoring them as tradition dictates.

An interesting final observation from our study is the relatively scarce number of comments from students on virtual or online components of a program. Our dataset reflected the first application cycle during the COVID-19 pandemic with radical paradigm shifts in away-rotation restrictions and students exploring virtual interview processes for the first time. Despite these unprecedented large-scale changes, only 4.2% of the total comments focused on the “virtual” aspect of program recruitment. This is in stark contrast to the significant amount of time spent by institutions and national organizations on virtual rotations, virtual tours, ongoing virtual interviews, virtual residency fairs, and virtual hangouts for students to socialize with residents. The data remains unclear based on this information from a single year to explain this lack of commentary. It may perhaps be due to lack of student participation in virtual rotations, given this was their first year being available as a rotation option, or perhaps virtual rotations were just simply not seen as appealing, thus demanding less discussion time on AOCs. Further analysis of subsequent years would be needed to fully analyze the effectiveness of virtual options for student applicants.

## LIMITATIONS

Potential limitations rest largely on data fidelity.^2^ ^,^ ^3^ ^,^ ^5^ Prior studies have also shown that AOC commenters may not necessarily reflect the entire applicant population.^6^ There is limited-to-no demographic information provided on the analyzed AOC. Additionally, the 183 narratives analyzed from the spreadsheet are relatively low compared to the number of applicants ranking EM as their preferred specialty or the 273 EM programs in existence at the time of 2021 NRMP Match.^21^ This may have put our analysis at risk of not reaching thematic saturation. Nevertheless, based on our collective experiences as EM residency applicants and as EM application advisers, we did not find any identified sub-themes particularly surprising or controversial. Although only a single AOC was analyzed in this study, we believe it to be fairly representative of commonly recurring student opinions and observations. For the purpose of this study, we specifically selected two sub-pages with the highest density of meaningful commentary for analysis; there is the potential that comments from other pages may reveal further themes or sub-themes.

## CONCLUSION

Our qualitative analysis of a single anonymous online community revealed six major themes discussed among students with regard to EM residency programs: working conditions; interpersonal relationships; learning experience; living conditions; postgraduate readiness; and online/virtual supplement. Most of the sub-themes to these categories represented aspects of clerkships and residency programs that are potentially modifiable by the program leadership. These findings suggest that AOC narratives cover several topics that may serve as useful external feedback for EM residency programs or clerkships. Iterative review of program-specific AOC narratives could serve as additional data in determining whether a program’s internal improvement efforts are noticed by students. Additional studies may help characterize the level of interest by key program stakeholders to consider and make changes based on feedback from AOC sources.

## Supplementary Information




