# Supplementary material for: Analysis of Anonymous Student Narratives About Experiences with Emergency Medicine Residency Programs
Source: West J Emerg Med. 2024 Feb 5;25(2):191–6. doi: 10.5811/westjem.17973 (PMC11000561; doi:10.5811/westjem.17973)
Supplement: Supplementary file 1 [file wjem-25-191-s001.docx]

**Appendix 1.** Codebook.

| Themes | Sub-themes | # | Total of coded comments |
| --- | --- | --- | --- |
| Working conditions | Patient population (underserved, volume, trauma, pathology etc) | 66 | 324 (38.5%) |
|  | Practice setting (community, academic, county, Lvl 1, HCA, etc.) | 66 |  |
|  | Perks (funding for travel/activities, food, lounge, parking, etc) | 37 |  |
|  | DEI (includes LGBTQ+) | 27 |  |
|  | Relationship with other specialties | 23 |  |
|  | Program reputation/prestige/age | 21 |  |
|  | Wellness | 20 |  |
|  | Work hours | 17 |  |
|  | Ancillary healthcare staff | 15 |  |
|  | EHR | 12 |  |
|  | Salary | 9 |  |
|  | Metrics | 6 |  |
|  | Moonlighting | 3 |  |
|  | Scutwork | 2 |  |
| Inter-personal relationships | Residents | 76 | 248 (29.5%) |
|  | Other leadership / faculty personality | 76 |  |
|  | PD personality | 56 |  |
|  | Responsiveness to upward feedback | 16 |  |
|  | Opportunity for upward feedback | 14 |  |
|  | Generic | 8 |  |
|  | Objective experience | 2 |  |
| Learning experience | Procedures | 25 | 118 (14.0%) |
|  | Didactics / conference | 20 |  |
|  | On-shift teaching | 17 |  |
|  | Autonomy | 16 |  |
|  | POCUS | 12 |  |
|  | Pediatric training | 12 |  |
|  | EMS/prehospital training | 5 |  |
|  | Scholarly tracks | 5 |  |
|  | Research | 5 |  |
|  | Personal patient load | 1 |  |
| Living conditions | Geography | 53 | 70 (8.3%) |
|  | Amenities | 11 |  |
|  | Cost of living | 6 |  |
| Post-graduate readiness | Fellowships | 17 | 46 (5.5%) |
|  | Jobs | 13 |  |
|  | PGY4 experience (length of training) | 12 |  |
|  | PGY3 experience (length of training) | 4 |  |
| Online/virtual supplement | Virtual interview day | 28 | 35 (4.2%) |
|  | Virtual tour | 4 |  |
|  | Virtual rotation | 2 |  |
|  | Website | 1 |  |

*HCA*, ; *DEI*, diversity, equity, inclusion; *EHR*, electronic health record; *PD*, program director; *POCUS*, point-of-care ultrasound; *EMS*, emergency medical services; *PGY*, postgraduate year.
